# Neuroethics, confidentiality, and a cultural imperative in early onset Alzheimer disease: a case study with a First Nation population

**DOI:** 10.1186/1747-5341-8-15

**Published:** 2013-10-16

**Authors:** Shaun Stevenson, B Lynn Beattie, Richard Vedan, Emily Dwosh, Lindsey Bruce, Judy Illes

**Affiliations:** 1 National Core for Neuroethics, Division of Neurology, Department of Medicine, The University of British Columbia, 2211 Wesbrook Mall, Koerner S124, Vancouver, BC, Canada; 2 Division of Geriatric Medicine, Department of Medicine, The University of British Columbia, Vancouver, BC, Canada; 3 School of Social Work, The University of British Columbia, 2080 West Mall, Vancouver, BC, Canada; 4 Clinic for Alzheimer Disease and Related Disorders, UBC Hospital 2211 Wesbrook Mall, Vancouver, BC, Canada

**Keywords:** Confidentiality, Global health neuroethics, Biomedical ethics, First nations, Research ethics, Early Onset Familial Alzheimer Disease, Community-based research

## Abstract

The meaningful consideration of cultural practices, values and beliefs is a necessary component in the effective translation of advancements in neuroscience to clinical practice and public discourse. Society’s immense investment in biomedical science and technology, in conjunction with an increasingly diverse socio-cultural landscape, necessitates the study of how potential discoveries in neurodegenerative diseases such as Alzheimer disease are perceived and utilized across cultures. Building on the work of neuroscientists, ethicists and philosophers, we argue that the growing field of neuroethics provides a pragmatic and constructive pathway to guide advancements in neuroscience in a manner that is culturally nuanced and relevant. Here we review a case study of one issue in culturally oriented neuroscience research where it is evident that traditional research ethics must be broadened and the values and needs of diverse populations considered for meaningful and relevant research practices. A global approach to neuroethics has the potential to furnish critical engagement with cultural considerations of advancements in neuroscience.

## Introduction

In an interdisciplinary effort bridging the clinical neurosciences, ethics, and geriatric services, we are engaged in community-based research with a First Nation population in Canada, in which a large family carries a presenilin-1 (PS1) gene mutation leading to early onset familial Alzheimer Disease (EOFAD). Our primary research goals are to explore the intersections of Western knowledge, traditional teachings, and culturally specific understandings about EOFAD. Through the evolution of this work, we have been faced with and have addressed unexpected internal and external challenges related to community-wide confidentiality. The internal challenges involve the Nation’s competing interests to guard against the possibility of stigma that may be associated with a predisposition to an incurable neurodegenerative disease, while also championing the benefits of research participation in a manner that fosters autonomy and self-determination. The external challenges involve achieving comprehensive outreach to individuals at rural and dispersed regions of the country, varying biological and cultural definitions of family, and the interconnectedness of family, community, and Nation. Both the internal and external challenges are of scholarly and procedural importance to the research team and Nation, and of personal significance to the community members.

There are ample existing research principles and guidelines that underscore the importance of protecting Indigenous communities; however, their scope is limited and they do not fully address the practical and conceptual challenges of community confidentiality when engaged in community-based research with a First Nation population. The unique and precarious circumstances described here highlight the importance of continuously revisiting issues in confidentiality, community-engaged discovery in the protection of Indigenous peoples in research, and the importance of seeking solutions that are mutually beneficial and supportive. Further, these challenges highlight the requirements for a global approach to neuroethics, which must strive to broaden traditional Western research ethics and grapple with these complex considerations of culture in neuroscience research.

### Neuroethics, culture and confidentiality

The intersections of neuroscience, neurotechnology, and society raise challenging ethical questions about how related developments may be perceived and utilized across cultures. The field of neuroethics addresses the ethical and legal issues of these innovations and their wide-ranging implications in the public sphere [1]. Whether defined as global [2], cosmopolitan [3], or pluralistic, as Giordano and Benedikter state, “neuroethics must be international, multi-cultural and multi-disciplinary, and not simply bound to philosophical dogma or defined by western ethical discourse” [1].

Through the advancement of a community-based research study about the experiences of a First Nations population with EOFAD, challenges of confidentiality have elucidated the need to broaden traditional Western understandings of research ethics and the integral role that neuroethics has in addressing these complex issues. While the case-study provided here offers only a limited scope of the ethical challenges in culturally relevant research, it will allow for further investigation into the complex interactions between research in neuroscience and the diverse populations affected by this research.

Issues of confidentiality in health research are wrought with concerns both important and complex, and research involving Indigenous^a^ populations that deals with confidentiality at the level of the community presents an array of unique challenges warranting additional attention. Confidentiality in health research has traditionally focused on the individual, with the patient/research participant at the center of ethical and privacy concerns [4]. The individual approach to ethics is highlighted in frameworks such as the *The Belmont Report,* which identifies “respect for persons, beneficence and justice” as the basic ethical principles guiding research on humans [5]. Genetics research has posed significant challenges to and expanded upon this individual focus on the protections of confidentiality, “since genetic information by nature is both individual and familial” [6].

Different still, is the role of confidentiality in community-based research involving identifiable populations, such as Indigenous communities. The need to protect an entire community, in addition to the individuals and families within the community, challenges the traditional limits of confidentiality. For example, New Zealand Maori culture operates within a tribal hierarchy in which individual rights, including the right to privacy, may be relinquished to maintain tribal structure [7]; the Maori culture emphasizes collectiveness, and even the ownership of genes and their mutations may be shared by the entire extended family, or *whanau*[7]. In Maori and other Indigenous populations, the Western emphasis on respect for personal autonomy and individual rights and risks may not adequately address the broader issues of community-based confidentiality or the integral role that these communities play in ensuring their own protection in research settings.

Research policies and guidelines primarily within Canada and Australia have been adapted to capture the ethical issues raised by the participation of Indigenous communities in human subjects research. Canada’s *Tri-Council Policy Statement 2 (TCPS2)* on “Research Involving the First Nations, Inuit and Métis Peoples of Canada,” for example, seeks to expand the scope of “Concern for Welfare,” and requires, “consideration of participants and […] their physical, social, economic and cultural environments, where applicable, as well as concern for the community to which participants belong” [8]. The *TCPS2*, and similar policy statements, such as those from the Canadian Institutes of Health Research and the National Health and Medical Research Council in Australia, acknowledge the integral role that Indigenous communities play in protecting the collective rights, interests and responsibilities that also serve the individual rights of community members [9,10].

Indigenous-specific research guidelines have emerged amidst a sullied history of research with Indigenous peoples – a history that further emphasizes the need for community confidentiality requirements for protecting identifiable populations in human subjects research [11]. Critics of research conducted in Indigenous communities have identified patterns of cultural insensitivity, lack of community involvement, stigma stemming from dissemination of results, lack of feedback during the research process, and exploitation of communities for academic or commercial gains, as just a few of the concerns that have plagued this research [4,5]. The Human Genome Diversity Project, for example, came under fire for its attempt to patent Indigenous peoples’ genetic materials, in many cases without their consent or under the guise of other research objectives [12]. A more explicit misconduct of research with Indigenous peoples was demonstrated when blood destined for rheumatic disease research from the Nuu-chah-nulth First Nation in British Columbia was used to investigate ancestry and population genetics. The Nations’ genetic materials were essentially treated as the researchers’ property, and became the fodder for hundreds of unrelated academic publications without the Nation’s consent or knowledge [13].

In discussing the philosophical and pragmatic challenges of protecting communities in research, Weijer writes, “Autonomous communities have their own politics, beliefs, and values and research may affect any of these elements” [14]. Indigenous communities have been affected negatively by research on many fronts, and beyond the mandated policies and guidelines already mentioned, a number of research principals, or codes of conduct, have been developed to ensure that work with Indigenous populations is done in a “good way” [15]. The National Aboriginal Health Organization in Canada promotes the principles of “OCAP”, ensuring that Indigenous populations maintain Ownership, Control, Access and Possession of various or all aspects of research within their communities [16]. Further notions of security and inclusion include the guiding principals of protection, participation and partnership [17], and “The 4R’s of Aboriginal Health:” respect, relevance, reciprocity, and responsibility [18].

While it is true that “guidelines for the protection of aboriginal communities in research are prototypical and probably the most extensive elaboration of protections available” [19], there remain practical and conceptual challenges in enacting and maintaining the highest degree of community confidentiality when working on genetic, brain, and health research with a remote and dispersed First Nation population. The guidelines and protocols for Indigenous research discuss the consideration of community confidentiality or anonymity in brief, stating simply that it should be a consideration where stigma is a concern, and that it should be determined prior to the commencement of research [8,9]. It is evident from our study that the current scope of Western research ethics is not always sufficient for engaging in research with culturally diverse, or non-Western populations.

The following case study presents some of the pragmatic and conceptual challenges of ensuring community confidentiality when conducting health research with a remote and dispersed First Nation population that is at risk for a familial neurodegenerative disease. These challenges highlight the need for a culturally nuanced neuroethics and broadened cultural approaches to research in neuroscience.

### Context

Between 1998 and 2009, nine members of a First Nations kindred were referred to the University of British Columbia Hospital Clinic for Alzheimer Disease and Related Disorders (UBCH-CARD) for medical assessment in the context of a strong family history of early-onset dementia [20]. The family originates from a remote rural community, with members dispersed throughout British Columbia, the Yukon, and Alberta. Seven of these nine individuals received clinical diagnoses of possible or probable Alzheimer Disease (AD). Genetic testing initiated on an affected family member in 2006 identified a novel PS1 gene mutation, thereby confirming a diagnosis of early-onset familial AD (EOFAD) [21]. Review of the family history identified over 100 family members in direct lineage of affected individuals and at risk of inheriting this condition [20].

The identification of a PS1 mutation in this family raised concerns regarding dissemination of information and provision of clinical services (including neurological and neuropsychological assessments, and genetic counseling) given constraints posed by geography and funding and, at the same time, introduced potential research and educational opportunities. Through a “Family Day” organized at UBCH-CARD and a health fair held in the Nation’s territories in 2009, the Nation signaled its desire to pursue further exploration about the disease and embarked on a collaborative endeavour with the UBC National Core for Neuroethics and UBCH-CARD.

## Methods

The methodological framework for this project is community-based research, guided by an Indigenous approach [22]. The approach relies on an infrastructure that includes a Community Advisory Group made up of key members of the Nation’s governance and leadership, a Community-based Researcher whose role is to actively facilitate research between the Nation and UBC team, and Community Liaisons who assist in the recruitment and organization of focus groups and interviews in the given territories. Every step of the research is filtered through and ushered by the continuous and dynamic interaction of the UBC team and these key representatives of the Nation.

The primary data for this project are cultural concepts and understandings of wellness and disease in aging and dementia collected through focus groups and interviews with members of the Nation. Issues of confidentiality have arisen during every phase of these research objectives.

### Findings and discussion

#### Sources of concern - confidentiality and community consent

Concerns about community confidentiality were captured early in the research process and reflected in the Research Agreement signed between the Nation and the research institution. The primary concerns were the possibility of stigma and stereotyping of individuals, families, and the community, and potential discrimination from employers and insurance companies. While these issues are not uncommon in research related to genetic diseases, they may be further amplified when working with Indigenous populations, as research occurs “amidst a historical context of cultural repression and reduced standards of health care availability” [23]. Furthermore, Indigenous communities have notions of family beyond the traditional biological model, where concepts such as “all my relations” may be used to explain the interdependency between individuals, family, community and Nation [24]. While genetic diseases such as EOFAD bring the biological definition of family to the fore, community-based research must understand cultural definitions of family at both a community and individual level.

Individuals, families, and the First Nation community are all affected by the EOFAD mutation under study. While, as Marshall and Berg are careful to emphasize, “community approval does not replace the need for individual consent” [25], in accordance with the practices of Indigenous research cited above, the Nation and its representatives have played a key role in consenting to research done within the community. The complex and integral ties between individual, family and community raise significant questions: What is the effect of maintaining or not maintaining community-wide confidentiality on families and individuals suffering from the disease? How does individual knowledge of the disease affect the community as a whole? How might the research affect families and individuals previously unaware of the genetic risk for the disease?

Upon consideration of these and other questions, the Research Agreement ensures that every effort is made to maintain the anonymity of the Nation in any publications or presentations of the research endeavour and findings. All parties have agreed to revisit confidentiality throughout various stages of the research project. At present, it remains in place for the ongoing protection of the community.

#### Protecting or silencing?

Despite the overriding pressure to retain the anonymity of the Nation, some individual community members have signaled their discontent in not having the community named [26]. These contrasting concerns involve issues of self-determination and autonomy, especially in the face of a history of subjugation within poor research practice. Article 3 of the *United Nations Declaration on the Rights of Indigenous Peoples* states, “Indigenous peoples have the right to self-determination. By virtue of that right they freely determine their political status and freely pursue their economic, social and cultural development” [27]. Further, Indigenous peoples in Canada have constitutional protections of their right to maintain their identity and participate as collectives in Canadian society [11]. Not naming an Indigenous community in research challenges the implementation of these rights for some members of the community.

While the principles of OCAP are integral to conducting research with Indigenous peoples and can be understood as “self-determination applied to research” [28], the protections and autonomy that these principles instill may be complicated by the application of community confidentiality. Writing for the First Nations Centre at the National Aboriginal Health Organization, Schnarch states that, “OCAP is not a doctrine or a prescription. It is a set of principles in evolution,” and therefore must be applied in conjunction with the best interests of the community [28].

Community-wide confidentiality may challenge the opportunity for members of the Nation to champion AD research from an Indigenous perspective, and to be recognized for the research contributions of their community. Open dialogue and deliberation on the benefits and harms of community confidentiality are essential both to ensure adequate protections and to foster self-determination through the evolving and adaptable principals of OCAP. Further, attention to and understanding of culture and the imperative role of a cultural community in the evolution of the research process are fundamental to conducting culturally-oriented research in neuroscience, and for ensuring that research is both inclusive and culturally relevant [1]. This type of community-based research must foster an approach to neuroethics that remains responsive, pluralistic and adaptable to the requirements of the community.

#### Confidentiality and knowledge transfer

Given the geographic distribution of this First Nation population, comprehensive outreach and dissemination of progress and results is a continuous obstacle requiring strategic initiatives. The Nation’s traditional territories are remote, with individuals living in rural, northern communities, and dispersed throughout various regions of the surrounding cities and provinces.

Traditional academic communications are often inadequate for keeping even urban-based communities up-to-date on research findings, let alone remote and dispersed communities for which access to follow up is especially challenging. Results not returned to the community, or returned in inaccessible language, has been identified by many Indigenous Nations as a significant grievance regarding research conducted within their communities [28]. Strategic, innovative and creative methods must be embraced to find solutions to these tensions that are grounded in technological, geographic and social forces.

Alongside the integral knowledge transfer enabled by community-based researchers and liaisons, the Internet can be a useful tool for widespread communication and for overcoming the logistics of geography. It is not without limitation, however, and breaches of confidentiality due to open access and unregulated networking is a fundamental concern. Research updates, community-based job postings, and recruitment letters posted online all have the potential to link researchers, individuals, and communities unexpectedly and openly to the sensitive research done within the community. The problem is double-edged: specific information enables specific recruitment and tailored dissemination of information, but places confidentiality at risk; generalized postings protect confidentiality but their effectiveness in recruiting and providing meaningful updates are diminished and limited. While secure web pages are a solution, easy access is then compromised by the need for passwords and layers of administration. Social media platforms, such as private Facebook groups are also an alternative, but monitoring and security are again a significant challenge.

The geographic nature of this community presents unique challenges to ensuring community protections. Community, in this sense, does not necessarily refer to a geographically fixed population, but rather to a dispersed group of people who share a cultural background. Once again, engaging such a community requires strong partnerships and clear understanding of how such a community is defined. Western approaches to community protections, even those specifically related to identifiable populations, do not adequately address the concerns of an Indigenous community with a population that is located remotely, as well as widely dispersed. As Giordano and Benedikter (2012) have argued, “if neuroethics is to authentically represent a naturalistic orientation to human cognition, emotions and behaviors, then it is essential to appreciate the ways that [cultural] variables and differences are manifest, and … adopt a more dialectical approach” [3]. Thus, a global approach to neuroethics that is collaborative and culturally-informed will have the potential to address the unique challenge of community confidentiality within a non-Western population.

## Conclusion

Weijer, Gold and Emanuel write, “Codifying protections for communities in research is a dialectical process that will require addressing these issues by proposing and refining potential safeguards based on conceptual reflection and practical experience” [19]. Research with Indigenous peoples has significantly improved with regard to respect, protections, and principles of appropriate conduct, but guidelines must remain flexible and dynamic enough to iteratively incorporate reflection based on experience, and look to the First Nation populations and communities themselves for best practice initiatives. Aboriginal people are one of the fastest growing populations in Canada [29]. If scientific and technological advancement is to be oriented towards the public good, socio-cultural values and contexts must be considered and incorporated in order to reflect an increasingly diverse society. A global approach to neuroethics will ensure that cultural plurality in neuroscience research is recognized and addressed appropriately. The potential challenges of non-Western based practices should not be a deterrent to community-based health research, but rather a motivation for further commitment and dedication to the advancement of knowledge and the alleviation of suffering and disease in a manner that is both inclusive and culturally relevant.

## Endnote

^a^We use the term *Indigenous* to refer to the First peoples of North America, Australia and New Zealand. Within Canada, these populations include First Nations, Métis and Inuit peoples. We use the term *First Nation* when referring to the specific Indigenous population with which we are working. The term *Indigenous* is akin to *Aboriginal* or *Native American.*

## Competing interests

The authors declare that they have no competing interests.

## Authors’ contributions

Concept and design: SS, JI, BLB. Collection and assembly of the data: JI, SS, BLB, LB. Data analysis and interpretation: SS, JI, BLB, ED, RV, LB, Drafting of the article: SS, JI, BLB. Critical revision of the article: BLB, JI, RV, ED, LB, SS. Final approval of the article: all authors. Obtaining of funding: JI, BLB. All authors read and approved the final manuscript.

## Authors’ information

SS is the Project Director on Cross Cultural Understandings of Aging and Dementia at UBC’s National Core for Neuroethics.

BLB is Professor Emeritus, Division of Geriatrics, Department of Medicine, UBC.

RV, Secwepemc First Nation, is Associate Professor, School of Social Work, UBC.

ED is a genetic counsellor at the UBC Hospital Clinic for Alzheimer Disease and Related Disorders and Clinical Instructor in the UBC Faculty of Medicine, Department of Medical Genetics.

LB is a PhD student in the Interdisciplinary Studies Graduate Program and National Core for Neuroethics, UBC.

JI is Canada Research Chair in Neuroethics, Director of the National Core for Neuroethics, and Professor of Neurology, Division of Neurology, Department of Medicine, UBC.
